# Supplementary material for: Encapsulated Nanodroplet Crystallization of Organic-Soluble Small Molecules
Source: Chem. 2020 Jul 9;6(7):1755–65. doi: 10.1016/j.chempr.2020.04.009 (PMC7357602; doi:10.1016/j.chempr.2020.04.009)
Supplement: Document S1. Supplemental Experimental Procedures and Figures S1–S3 [file mmc1.pdf]

Chem, Volume *6*

Supplemental Information

Encapsulated Nanodroplet Crystallization

of Organic-Soluble Small Molecules

Andrew R. Tyler, Ronnie Ragbirsingh, Charles J. McMonagle, Paul G. Waddell, Sarah E. Heaps, Jonathan W. Steed, Paul Thaw, Michael J. Hall, and Michael R. Probert

Figure S1: Additional compounds **S1-S4** for which single crystals were successfully grown *via* the ENaCt protocol and subsequently analysed by SCXRD. Molecular structures, electron density maps and refined crystallographic molecular models are shown. Each structure is provided with selected crystallographic information and ENaCt conditions for crystal growth.

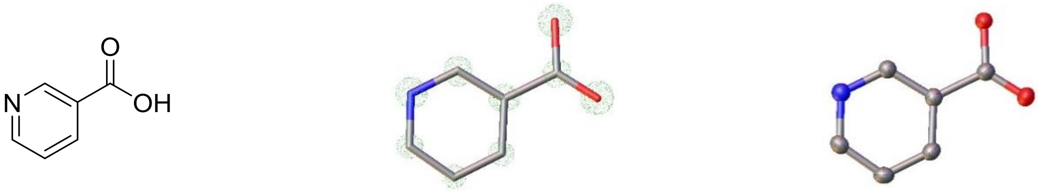

Nicotinic acid (**S1**): R_1 = 0.0594, ${}_{w}R_2$ = 0.1100, Residual e^- density ($e^-Å^3$) min = -0.231 and max = 0.1171
200 nL Mineral oil, 50 nL of 40 mg/mL of nicotinic acid in DMSO, 100 nL toluene.

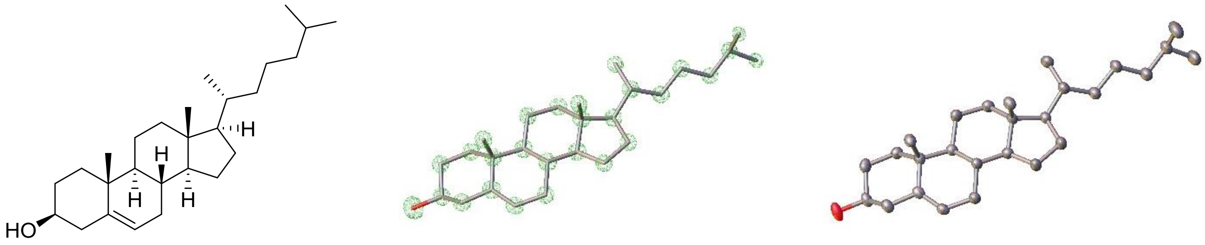

Cholesterol (**S2**): R_1 = 0.0594, ${}_{w}R_2$ = 0.1118, Residual e^- density ($e^-Å^3$) min = -0.162 and max = 0.199
200 nL FC-40, 50 nL of 25 mg/mL of cholesterol in DMF, 25 nL *n*-butanol.

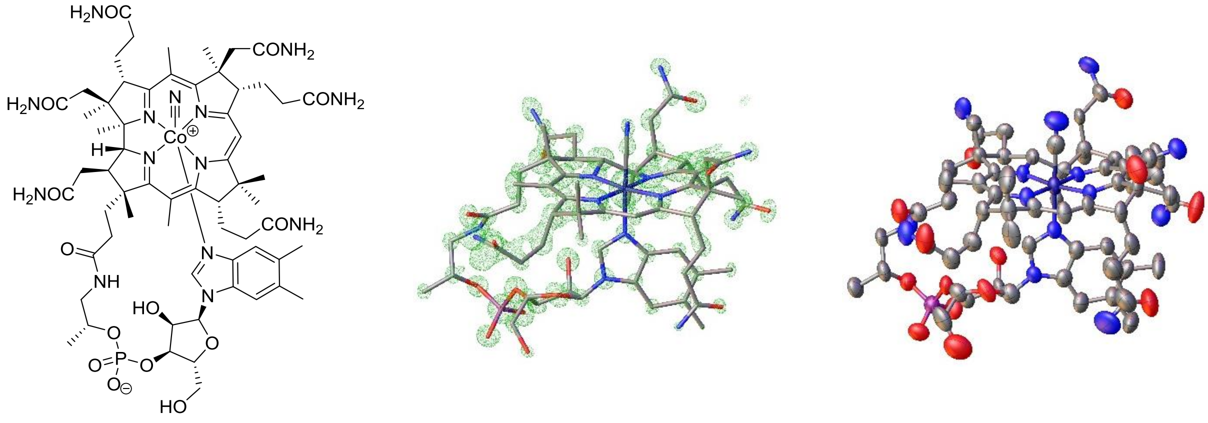

Vitamin B12 (**S3**): R_1 = 0.0902, ${}_{w}R_2$ = 0.1535, Residual e^- density ($e^-Å^3$) min = -0.433 and max = 0.260, Flack = 0.071(12)
200 nL Mineral oil, 50 nL of 50 mg/mL in DMSO, 50 nL (±)-2-methyl-2,4-pentanediol.

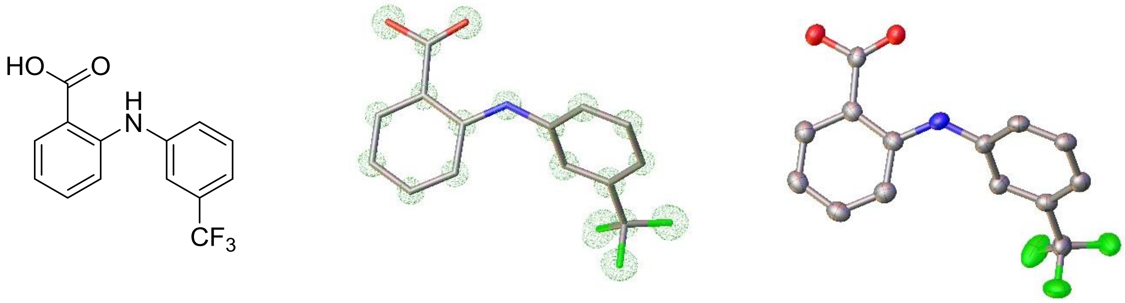

Flufenamic acid (**S4**): R_1 = 0.1093 , ${}_{w}R_2$ = 0.1776, Residual e^- density ($e^-Å^3$) min = -0.314 and max = 0.343
200 nL PDMSO, 50 nL of 50 mg/mL flufenamic acid in DMF, 50 nL H_2O.

Figure S2: Additional compounds **S5-S7** for which single crystals were successfully grown *via* the ENaCt protocol and subsequently analysed by SCXRD. Molecular structures, electron density maps and refined crystallographic molecular models are shown. Each structure is provided with selected crystallographic information and ENaCt conditions for crystal growth.

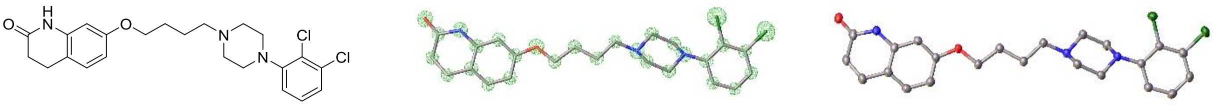

Aripiprazole (**S5**): $R_1 = 0.0599$, ${}_{w}R_2 = 0.0947$, Residual e^- density ($e^-Å^3$) min = -0.256 and max = 0.250
200 nL Fomblin Y, 50 nL of 20 mg/mL of aripiprazole in DMSO.

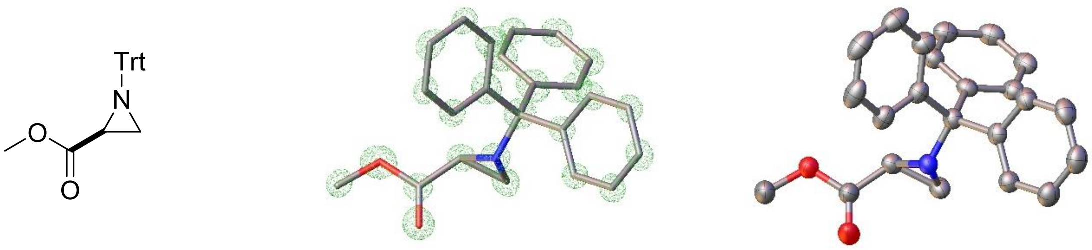

Methyl (*S*)-1-tritylaziridine-2-carboxylate (**S6**): $R_1 = 0.0461$, ${}_{w}R_2 = 0.0935$, Residual e^- density ($e^-Å^3$) min = -0.129 and max = 0.130, Flack = 0.0(2)
200 nL PDMSO, 50 nL of 40 mg/mL of methyl (*S*)-1-tritylaziridine-2-carboxylate in DMF, 100 nL H_2O.

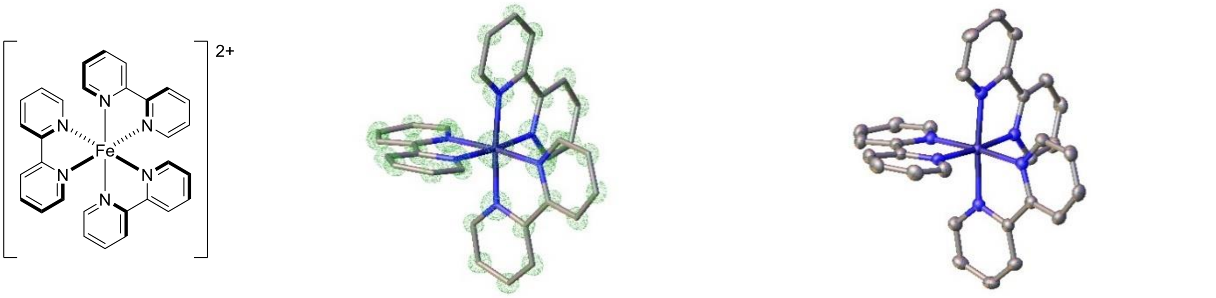

$[Fe(bipy)_3]^{2+}\cdot 2PF_6^-$ (**S7**): $R_1 = 0.0331$, ${}_{w}R_2 = 0.0707$, Residual e^- density ($e^-Å^3$) min = -0.288 and max = 0.299
200 nL FC 40, 50 nL of 25 mg/mL of $[Fe(bipy)_3]^{2+}\cdot 2PF_6^-$ in DMSO (*Note:* counter ions omitted for clarity).

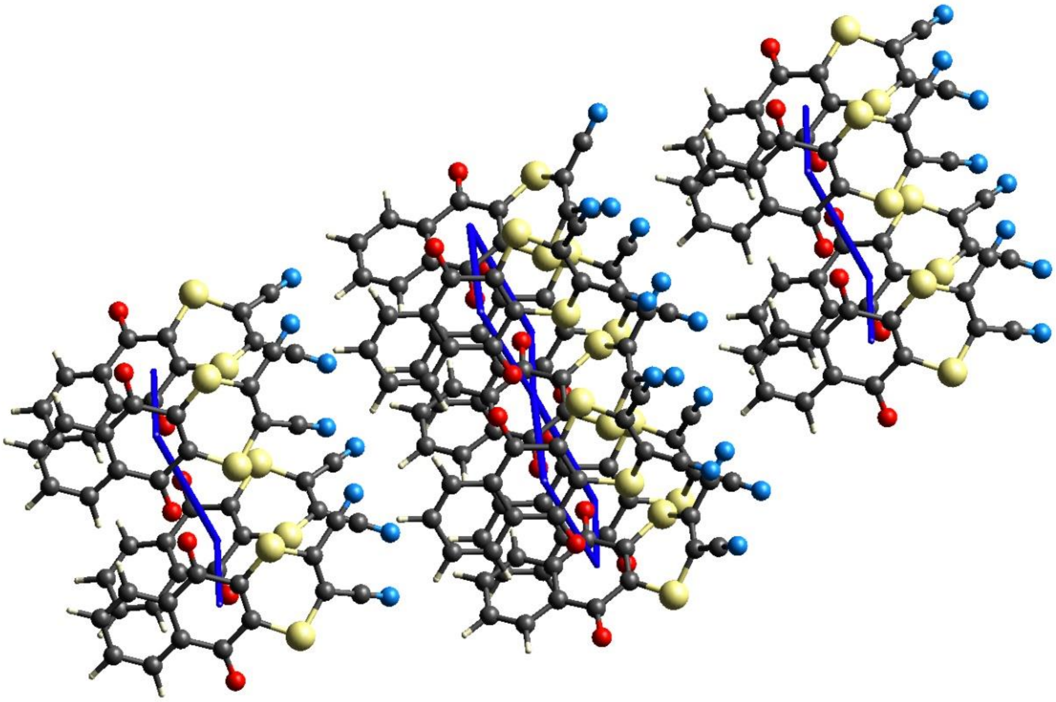

Figure S3: Total-energy-framework diagram for dithianon using cylinder size 50 for energies. Note energies less than 15 kJmol^{-1} have been omitted for clarity. Energies were calculated for the 150 K structure obtained by single crystal X-ray diffraction using the B3LYP/6-31G(d,p) functionals.

Supplemental Experimental Procedures

Materials.

Commercially available oils and chemical substrates were purchased from Sigma Aldrich, Fluorochem, Alfa Aesar or Molecular Dimensions.

5-Methyl-2-[(2-nitrophenyl)amino]-3-thiophenecarbonitrile (ROY) was provided by J.W. Steed, metal complex ($[Fe(bipy)_3]^{3+}$) was provided by M.R. Probert, methyl (*S*)-1-tritylaziridine-2-carboxylate was provided by A.R. Tyler, and methyl 4-(5,5-difluoro-5*H*-4λ^4,5λ^4-dipyrrolo[1,2-*c*:2',1'-*f*][1,3,2]diazaborinin-10-yl)benzoate was provided by M.J. Hall.

Laminex™ glass 96 well plates with a 100 micron spacer and cover slips were purchased from Molecular Dimensions. Glass coated 96 well plates were obtained from Fisher Scientific.

Sample preparation

Stock solutions of each substrate were freshly prepared for each crystallisation experiment. Samples were weighed into a screw top vial, dissolved in a suitable solvent at the desired concentration and then warmed where appropriate (40 °C, 30 min) to ensure that all solids were dissolved. Stock solutions were left to cool (1 hour, r.t.) and assessed *via* optical microscopy to ensure premature crystallisation had not occurred before being used in any ENaCt experiment.

Crystallisation of substrates

Crystallisation experiments were completed using a TTP LabTech mosquito® liquid handling robot using LAMINEX™ glass 96 well plates with a 100 micron spacer and sealed with a glass cover slip. An appropriate volume (typically 200 mL) of each oil was first dispensed onto a LAMINEX™ plate (aspirate 1.0 mm/min, dispense 1.0 mm/min). After which 50 nL of substrate solution was injected into each oil droplet (aspirate 20 mm/min, dispense 20 mm/min), followed by an additional injection of a secondary solvent if required (aspirate 20 mm/min, dispense 20 mm/min). Plates were then sealed with a glass cover slip, stored in the dark at room temperature and inspected for crystal growth at regular intervals. Visualisation of the experiment wells was carried out with a Nikon SMZ1000 microscope fitted with a cross polariser. Photographs were taken with a GXCAM-U3-5 5.1MP camera.

Single crystal X-ray diffraction studies

Upon observation of suitable crystals, wells were opened, with use of a tungsten carbide scriber to remove a small portion of the glass cover slide, and the crystal manipulated using Mitegen Kapton microtools. Crystals were transferred to a glass slide and extracted under oil (Fomblin YR-1800) with a standard Mitegen Kapton loop, and mounted onto either an in-house diffractometer (Rigaku Oxford Diffraction Xcalibur, Atlas, Gemini ultra, or Bruker D8 Vantage, Photon 2, dual Incoatec IµS (Ag/Cu) both equipped with an Oxford Cryosystems Cryostream open-flow cooling device and maintained at 150 K) or stored in a liquid N_2 storage dry shipper for investigation on beamline I19 of Diamond Light Source, *via* remote access.

Ordinal logistic regression

Let the outcome of each experiment be encoded by an ordinal variable Y where:

Y = {1, if no solid (still solvated); 2, if phase separation; 3, if solid, but not crystalline; 4, if small crystal; 5, if large crystal.

A proportional odds model for ordinal logistic regression was used to assess the relationship between the response variable Y and covariates, which comprised two quantitative variables (volume of solvent and volume of oil) and three categorical variables (solvent, type of oil and compound). Inference was performed in the Bayesian framework, carrying out the necessary numerical computation using rstan[S1], the R interface to the Stan software[S2]. Use of FC40 and, in particular, PDMSO both appear to have a positive relationship with the response variable; the posterior probabilities that the corresponding regression coefficients are greater than zero, indicating positive association, are each equal to 1.00, to two decimal places.

Supplemental References

S1. Stan Development Team (2016). RStan: the R interface to Stan. R package version 2.12.1.

S2. Carpenter, B., A. Gelman, M. D. Hoffman, D. Lee, B. Goodrich, M. Betancourt, M. A. Brubaker, J. Guo, P. Li and A. Riddell (2017). Stan: A probabilistic programming language. *Journal of Statistical Software* 76 (1), 1–32.
