# Supplementary material for: Encapsulated Nanodroplet Crystallization of Organic-Soluble Small Molecules
Source: Chem. 2020 Jul 9;6(7):1755–65. doi: 10.1016/j.chempr.2020.04.009 (PMC7357602; doi:10.1016/j.chempr.2020.04.009)
Supplement: Document S2. Article plus Supplemental Information [file mmc3.pdf]

Chem

CellPress

Article

Encapsulated Nanodroplet Crystallization of Organic-Soluble Small Molecules

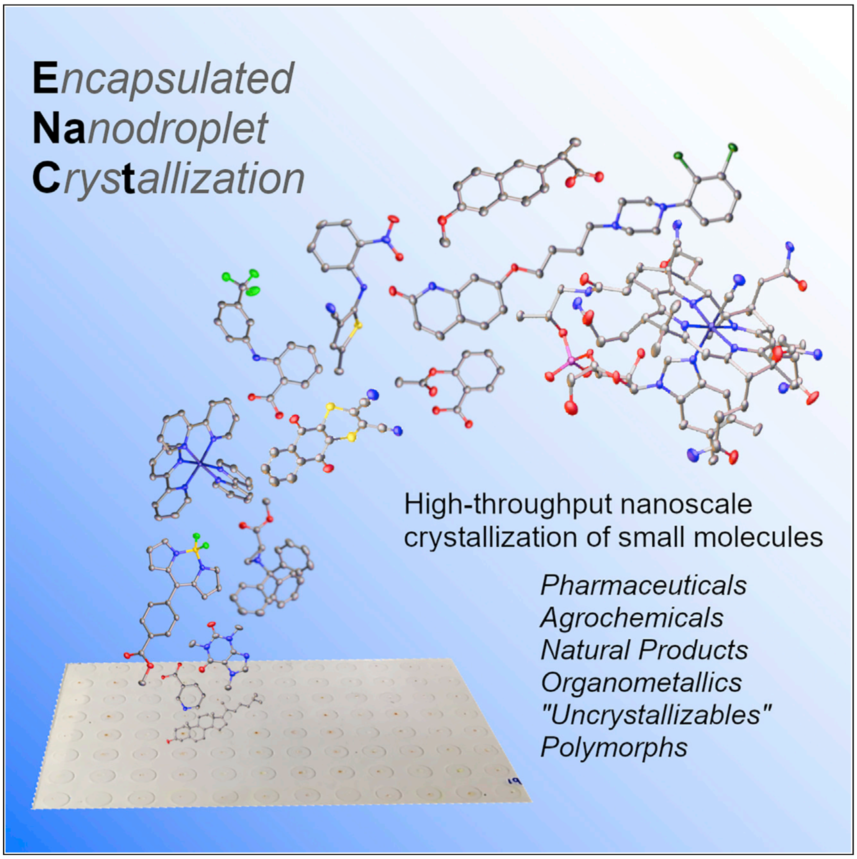

The routine crystallization of small molecules for single-crystal X-ray analysis remains a considerable experimental challenge. We report a general method for the high-throughput nanoscale crystallization of organic-soluble small molecules: encapsulated nanodroplet crystallization (ENaCt). ENaCt provides crystals suitable for X-ray analysis, allowing structural and *de novo* absolute stereochemical assignment for a diverse and challenging range of small molecules (bioactives, natural products, organometallics, etc.), as well as acting as a tool for new polymorph discovery.

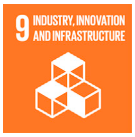

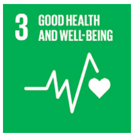

Andrew R. Tyler, Ronnie Ragbirsingh, Charles J. McMonagle, ..., Paul Thaw, Michael J. Hall, Michael R. Probert

michael.hall@ncl.ac.uk (M.J.H.)
michael.probert@ncl.ac.uk (M.R.P.)

HIGHLIGHTS

Single crystals of small molecules are grown from nanoscale droplets of organic solvent

Discovery of the 13th polymorph (R18) of olanzapine precursor ROY

X-ray diffraction analysis of "uncrystallizable" agrochemical dithianon

Tyler et al., Chem 6, 1755–1765
July 9, 2020 © 2020 The Authors. Published by Elsevier Inc.
https://doi.org/10.1016/j.chempr.2020.04.009

Chem

Article

Encapsulated Nanodroplet Crystallization of Organic-Soluble Small Molecules

Andrew R. Tyler,[1] Ronnie Ragbirsingh,[1] Charles J. McMonagle,[1] Paul G. Waddell,[1] Sarah E. Heaps,[2] Jonathan W. Steed,[3] Paul Thaw,[4] Michael J. Hall,[1,5,*] and Michael R. Probert[1,*]

SUMMARY

Single-crystal X-ray diffraction analysis (SCXRD) constitutes a universal approach for the elucidation of molecular structure and the study of crystalline forms. However, the discovery of viable crystallization conditions remains both experimentally challenging and resource intensive in both time and the quantity of analyte(s). We report a robot-assisted, high-throughput method for the crystallization of organic-soluble small molecules in which we employ only micrograms of analyte per experiment. This allows hundreds of crystallization conditions to be screened in parallel with minimal overall sample requirements. Crystals suitable for SCXRD are grown from nanoliter droplets of a solution of analyte in organic solvent(s), each of which is encapsulated within an inert oil to control the rate of solvent loss. This encapsulated nanodroplet crystallization methodology can also be used to search for new crystal forms, as exemplified through both our discovery of a new (13th) polymorph of the olanzapine precursor ROY and SCXRD analysis of the "uncrystallizable" agrochemical dithianon.

The Bigger Picture

Small molecules can form crystalline solids, in which individual molecules pack together into ordered three-dimensional arrays. Once a suitable crystal is grown, the packing and atomic connectivity of the constituent molecules can be studied by X-ray diffraction. However, the discovery of experimental conditions for successful crystal growth is often challenging. We have developed a nanoscale crystallization technique for organic-soluble small molecules by using high-throughput liquid-handling robotics to undertake multiple crystallization experiments simultaneously with minimal sample requirements and high success rates. We showcase our methodology through the crystallization of a diverse set of small molecules, including "uncrystallizables," combined with structural analysis by X-ray diffraction. We anticipate that this rapid and reliable method for small-molecule crystallization will have far-reaching impact, facilitating academic and industrial research in the molecular sciences.

INTRODUCTION

Single-crystal X-ray diffraction (SCXRD) allows for the direct analysis of crystalline small molecules, providing structural information with sub-Ångstrom resolution,[1] *de novo* absolute stereochemistry assignment (via anomalous dispersion),[2] and detailed information on intermolecular interactions and structural packing motifs. Modern in-house single-crystal diffraction instrumentation (e.g., microfocus X-ray tubes, multi-layer focusing optics, and very-low-noise area detectors) allows for the routine investigation of crystals, containing only light atoms, with dimensions ≈ 50 μm.[3] That, in combination with improved access to synchrotron radiation sources (e.g., remote-access beamlines),[4] has allowed SCXRD to become a ubiquitous research technique for molecular analysis, given a suitable crystalline sample; as of 2019, over one million crystal structures have been deposited in the Cambridge Crystallographic Data Centre (CCDC).[5]

Most small molecules are capable of existing as crystalline solids either as pure materials or in conjunction with other species (e.g., salts, hydrates, solvates, or co-crystals)[6] and thus are theoretically amenable to SCXRD analysis. However, the growth of suitably sized, high-quality single crystals remains experimentally challenging in that researchers still rely on time-consuming manual methods (i.e., solvent evaporation, exchange, or diffusion experiments),[7] which typically take many weeks to complete and require milligrams of analyte per experiment.

Recently, a number of approaches have attempted to circumnavigate the problems associated with traditional small-molecule crystal growth while retaining the

This is an open access article under the CC BY license (http://creativecommons.org/licenses/by/4.0/).

analytical power provided by diffraction-based techniques. Fujita's "crystalline sponge" method relies on the long-range ordering of small organic "guest" molecules within a single crystal of a pre-prepared porous host, and subsequent SCXRD analysis of this host-guest complex provides structural information on the guest molecule.[8,9] However, as a result of weak host-guest interactions, the small-molecule guest must be carefully paired with an appropriate host, and the physical separation of guest molecules precludes analysis of any potential intermolecular interactions or other solid-state properties. Electron diffraction (e.g., MicroED) has subsequently emerged as a technique for the analysis of crystalline small molecules, where electron diffraction patterns are obtained from small single crystals (1–10 μm in each dimension).[10–12] However, the intensity of the electron beam causes rapid sample degradation via *in situ* radical generation; the crystals must be stable within the vacuum stage (precluding the study of hydrates and solvates), and the elucidation of absolute stereochemistry is far from routine (e.g., dynamical refinement).[13]

Thus, SCXRD remains the analytical technique of choice for the study of small molecules, although it is hampered by the practical constraints of crystal growth. Easy access to single crystals, suitable for SCXRD, would therefore be a significant enabling step across the molecular sciences. Solution-phase crystallization commences with nucleation from a supersaturated solution followed by crystal growth.[14] Nucleation is a stochastic process that, especially in the case of heterogeneous nucleation, is heavily influenced by the local environment (e.g., solvent[s], impurities, contact surfaces, and convection). Therefore, control is required over both the conditions of supersaturation and the number of nucleation sites present. The solid-state energy landscape of a molecule can be further complicated by the existence of multiple crystalline forms (e.g., salts, hydrates, solvates, co-crystals, or polymorphs). Hence, the discovery of successful crystallization conditions requires the exploration of large volumes of experimental space. Despite considerable research into the development of new small-molecule crystallization techniques, the current state of the art still requires the use of milligrams of analyte per experiment or is restricted to specific molecular classes, limiting the experimentally accessible envelope.[15–18]

Here, we discuss our use of high-throughput crystallization techniques as a general method for the growth of single crystals of organic-soluble small molecules on the nanoscale. Taking inspiration from "microbatch-under-oil" protein crystallization techniques,[19,20] our key enabling breakthrough involves the use of inert viscous oils to control the rate of solvent loss from nanoliter-scale droplets of organic solvent, each containing a few micrograms of small-molecule analyte. Oil encapsulation results in a slow increase in sample concentration up to and beyond the point of saturation, even for nanoliter-scale droplets of volatile organic solvents. When oil encapsulation is combined with the restricted number of nucleation sites available in such small droplets, we observe the growth of high-quality single crystals with dimensions $\geq$ 20 μm. These crystals are shown to provide excellent X-ray diffraction data sets on either in-house instrumentation or central facility beamlines.

We also show that encapsulated nanodroplet crystallization (ENaCt) experiments can be efficiently set up via a suitable liquid-handling robot, resulting in a semi-automated experimental approach in which hundreds of individual crystallization experiments can be initiated within a few minutes. This high-throughput parallel screening approach allows for rapid exploration of crystallization space and thus reliable access to suitable crystals. This is demonstrated here through the successful crystallization and SCXRD analysis of 14 structurally diverse molecules, including *de novo* absolute stereochemical analysis, polymorph discovery (including a hitherto

[1]Chemistry, School of Natural and Environmental Sciences, Newcastle University, Newcastle upon Tyne NE1 7RU, UK

[2]School of Mathematics, Statistics and Physics, Newcastle University, Newcastle upon Tyne NE1 7RU, UK

[3]Department of Chemistry, Durham University, Durham DH1 3LE, UK

[4]SPT Labtech, Melbourn Science Park, Melbourn, Hertfordshire SG8 6HB, UK

[5]Lead Contact

*Correspondence:
michael.hall@ncl.ac.uk (M.J.H.),
michael.probert@ncl.ac.uk (M.R.P.)

https://doi.org/10.1016/j.chempr.2020.04.009

unknown 13th polymorph of 5-methyl-2-[(2-nitrophenyl)amino]-3-thiophenecarbonitrile [ROY], R18), and the crystallization of "uncrystallizable" substrates (dithianon).

RESULTS AND DISCUSSION

Preliminary Oil-Encapsulated Nanodroplet Crystallizations

Nanoscale crystallizations are typically incompatible with the use of analyte solutions containing a high percentage of organic solvents because rapid solvent evaporation leads to deposition of the analyte as amorphous material. The rate of evaporative loss is proportional to the air-liquid interface surface area and is thus rapid in terms of percentage volume for a nanoliter-scale droplet.

We postulated that the evaporative loss from a nanodroplet of organic solvent could be slowed by reduction of the air-liquid interface surface area by encapsulation of said nanodroplet within a droplet of oil. This slower, more controlled concentration of the analyte would lead to improved crystal growth.

It should be noted that using oils to aid in the growth of single crystals for X-ray diffraction analysis from aqueous buffer solutions is known in protein crystallization[19,20] and has recently been applied to the crystallization of highly water-soluble salts of small organic molecules.[21] However, this "microbatch-under-oil" approach requires the use of aqueous solutions of water-soluble molecular analytes, typically in combination with low-density, mobile paraffin oils, to ensure phase separation while maintaining droplet mobility. These conditions are incompatible with most small organic molecular analytes because of their poor solubility in the aqueous buffers utilized, and the direct exchange of the aqueous buffer in such an experiment with an organic solvent would make the maintenance of phase separation challenging. Therefore, we chose to focus on using viscous oils with low miscibility in common organic solvents to encapsulate our organic nanodroplets, thus maintaining droplet integrity and encapsulation of the analyte solution.

Furthermore, the oils utilized would have to exhibit low vapor pressures to prevent evaporation and be chemically inert because of the anticipated long contact times with the organic solvent utilized. Thus, we undertook a series of preliminary oil-encapsulated nanodroplet crystallization experiments that involved using an SPT Labtech mosquito® liquid-handling robot to place 250 nL droplets of different test oils into a 96-well glass plate (Laminex or SWISSSCI LCP 100-micron), into which 50 nL of a solution of a small organic molecule (ROY) in an appropriate organic solvent was placed. ROY was chosen as a test substrate because of its propensity to form distinctively colored crystals, facilitating visual analysis of the crystallization process. The resulting 96-well plates were sealed (glass cover) and stored at room temperature (RT), and each well was examined periodically for crystal formation by polarizing light optical microscopy. After several days, we observed the formation of large numbers of crystals, many of which were single crystals of sufficient dimensions for SCXRD (Figure 1), providing the initial experimental support for our research postulate (see Methods Video S1).

Comparison of Encapsulated versus Non-encapsulated Nanodroplet Crystallizations

On the basis of these preliminary results, we attempted to further validate our experimental design through a comparison of organic-solvent-based nanodroplet crystallization conditions with and without oil encapsulation by employing a set of five representative small-molecule analytes: aspirin (**1**), caffeine (**2**), BODIPY (methyl 4-(5,5-difluoro-5*H*-4λ,^{4}5λ^4-dipyrrolo[1,2-*c*:2′,1′-*f*][1,3,2]diazaborinin-10-yl)benzoate) (**3**), (*R*)-BINOL (**4**), and

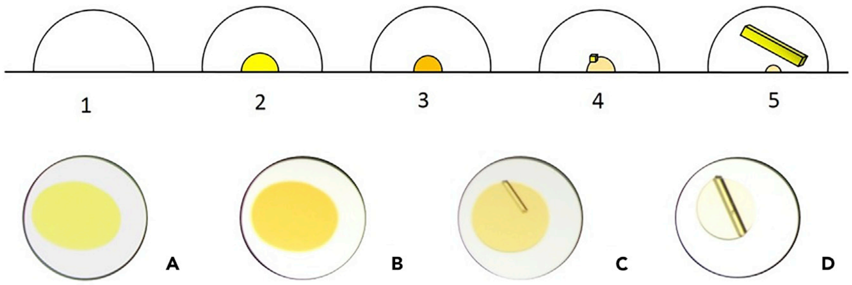

Figure 1. Cross-Section Schematic of an ENaCt Experiment (Top) and ENaCt Experiment with 200 nL Mineral Oil and 50 mg/mL ROY in DMSO (Bottom).
(1) Viscous inert oil dispensed onto a well of a 96-well glass plate, (2) solution of analyte in organic solvent injected into an oil droplet, (3) evaporative solvent loss to supersaturation, (4) nucleation, and (5) crystal growth. (A) solution of solvated analyte under oil, (B) evaporative solvent loss to supersaturation, (C) onset of crystal growth, and (D) complete crystallization.

(*S*)-naproxen (**5**). An SPT Labtech mosquito® liquid-handling robot was used to dispense the first two test oils (50–300 nL of Fluorinert FC-40 and polydimethyl siloxane [PDMSO]) on a 96-well glass plate (Laminex or SWISSCI LCP100-micron) and then dispense 50 nL of analyte solution in an organic solvent (40–50 mg/mL) either into the oil droplet or directly onto the plate. Six solvents were chosen—acetone, ethyl acetate, ethanol, 1,2-dichloroethane, *N*,*N*-dimethylformamide, and dimethyl sulfoxide—because they span a range of boiling points (bp = 56°C, 77°C, 79°C, 84°C, 153°C, and 189°C) and polarities (ε = 21, 6, 35, 11, 38, and 46). For control experiments employing no oil, we added additional solvent to give a total volume of 100–350 nL to maintain a comparable droplet size with oil-encapsulated experiments and thus a similar solvent-air interface area. After the experimental setup of the nanodroplets, the crystallization plates were partially sealed from atmospheric conditions with the use of a glass cover and stored at RT (see Supplemental Experimental Procedures). After 1, 3, 7, and 14 days, we photographed and assessed individual wells by optical, cross-polarizing light microscopy to establish the presence of single crystals. We chose 14 days as a common end point for the experiments to provide a balance between crystal formation and complete desolvation of the sample. Experimental outcomes were categorized on a 1–5 scale (1 = no solid [still solvated]; 2 = phase separation; 3 = solid [amorphous or microcrystalline]; 4 = small single crystals [<50 μm]; and 5 = large single crystals [>50 μm]) (Figure 2).

After 14 days, the majority of experiments had reached an end point (2–5). Visual inspection of the results showed that those crystallization conditions employing low-boiling-point solvents (acetone, ethyl acetate, and ethanol) without oil encapsulation had only given amorphous or microcrystalline solids resulting from the anticipated rapid evaporative solvent loss. Higher boiling solvents (1,2-dichloroethane, dimethylformamide [DMF], and dimethyl sulfoxide [DMSO]) without oil encapsulation did result in small numbers of single crystals (e.g., **4** in 1,2-dicholorethane), and some samples remained in solution (e.g., DMSO). However, as a consequence of more controlled solvent loss, oil encapsulation with both fluorous (FC-40) and non-fluorous (PDMSO) oils improved the experimental outcomes for all solvents examined such that many more small (4) and large (5) single crystals were observed. Interestingly, the oil encapsulation of nanodroplets of DMSO analyte solutions improved experimental outcomes, whereas non-encapsulated samples remained in solution. This suggests that in the case of oil-encapsulated DMSO nanodroplets, an additional route to crystal formation might be occurring (e.g., diffusional loss of solvent into the oil or nucleation at the solvent-oil interface).

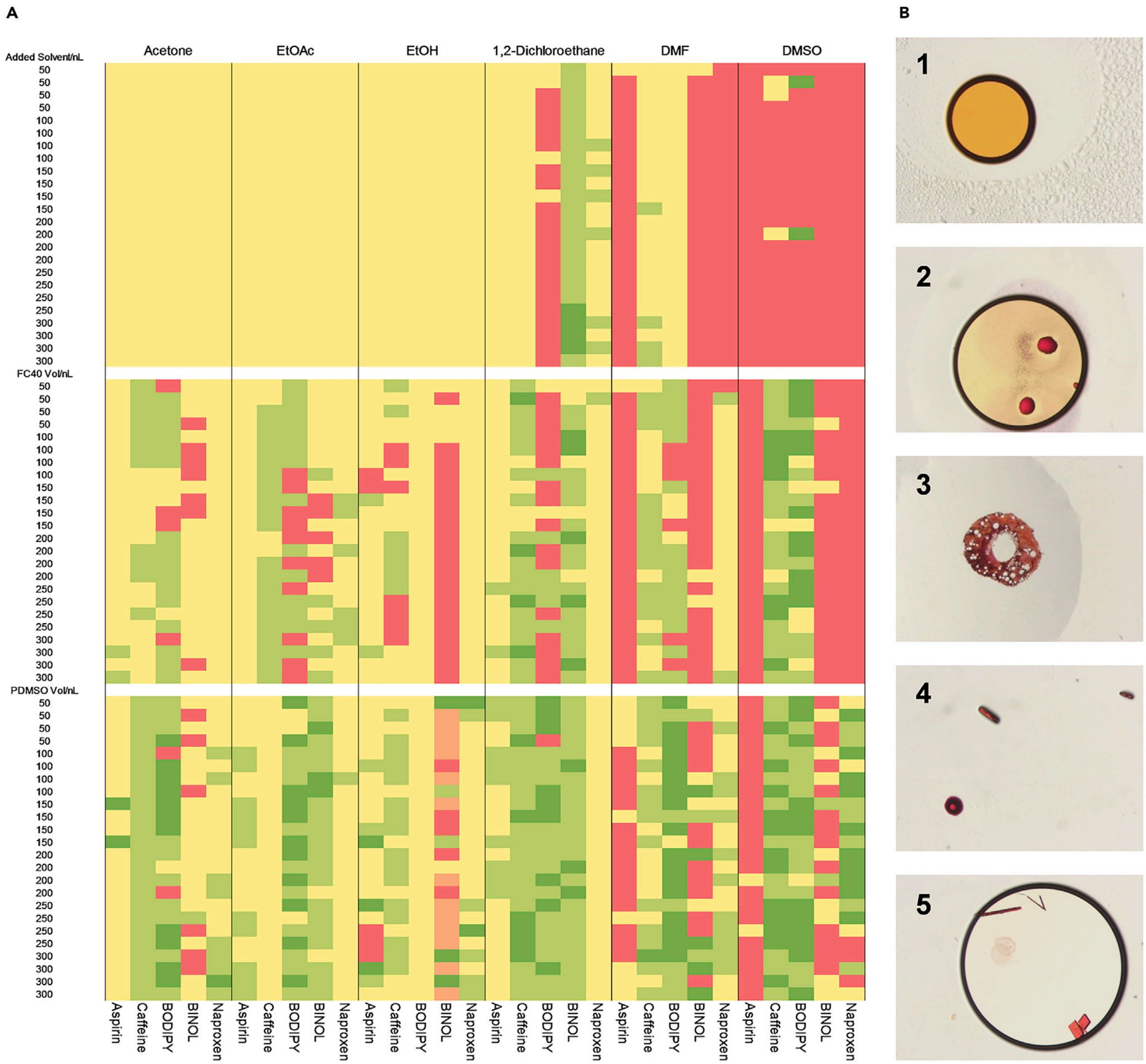

Figure 2. Encapsulated versus Non-encapsulated Nanodroplet Crystallizations

(A) Crystallization conditions for analytes **1**–**5** dissolved in organic solvents (acetone, ethyl acetate, ethanol, 1,2-dichoroethane, dimethylformamide, or dimethyl sulfoxide) with and without oil encapsulation (FC-40 or PDMSO). (1) Red: sample remains in solution; (2) orange: phase separation from solution and no solid; (3) yellow: amorphous or micro-crystalline solids; (4) light green: small single crystal(s); (5) dark green: large single crystal(s).
(B) Representative experimental outcomes (1–5) shown with BODIPY (**3**) for ease of visualization.

To gain further insight into the impact of oil encapsulation on small-molecule crystallization, we performed a statistical analysis of the experimental outcomes. We fitted a proportional odds model for ordinal logistic regression in the Bayesian framework to access the relationship between the response variable (experimental outcomes 1–5) and the covariates (volume of solvent, volume of oil, type of solvent, type of oil, and molecule).[22] The use of oils, both FC-40 and PDMSO, showed a clear positive relationship with the outcome of the crystallization experiments such that more suitable single crystals were formed under oil-encapsulation conditions (see Supplemental Experimental Procedures).

Finally, suitable single crystals of each of the five compounds (**1**–**5**) were retrieved from their corresponding 96-well plates, mounted, and analyzed by SCXRD. In all cases, high-quality data were recorded to a minimum completeness of 99% at a minimum resolution of 0.84 Å on standard in-house diffractometers using Cu Kα X-radiation (λ = 1.54184 Å). Molecular structures were then obtained through structure solution and refinement using the OLEX2[23] interface to the SHELX[24] suite of programs. Furthermore, the absolute stereochemical assignments were confirmed by successful refinement of the Flack parameter derived from anomalous dispersion measurements for both **4** and **5** (Figure 3).

To further validate the capability of the ENaCt protocol, we subsequently applied it to a broader set of chemical compounds (**S1**–**S7**). These experiments were further refined through the addition of small volumes (up to 100 nL) of a secondary solvent to the solution of analyte within the inert oil droplet and the use of a wider range of inert oils, allowing an expansion of the protocol's experimental space-sampling capability through the fine-tuning of crystallization conditions. In all cases, suitable single crystals were successfully grown and subsequently analyzed by SCXRD, resulting in full structure solution and refinement coupled with absolute stereochemical assignments where appropriate (Figures S1 and S2).

Polymorph Screening of ROY

After the success of our previous crystallization experiments, we applied our ENaCt protocol to a well-known problem in solid-state chemistry, namely polymorphism. This is the phenomenon where a given compound crystallizes into different solid phases—differing only by the three-dimensional arrangement of molecules in space—that return to indistinguishable solution or liquid states.[25] The discovery of polymorphic forms is particularly suited to our ENaCt protocol because large numbers of crystallization experiments can be undertaken in parallel (reducing the total number of person hours required) with well-defined yet different crystallization conditions.

Polymorphism is particularly relevant to the pharmaceutical industry because different polymorphs of a compound can have significantly different physical properties (e.g., solubility and stability). This is a particular concern for the preparation of active pharmaceutical ingredients (APIs), where such physical properties directly affect the bioavailability of a compound. Thus, the early identification of accessible polymorphs of an API is of significant economic importance because an unexpected appearance of a previously unknown stable polymorphic phase can result in temporary market withdrawal pending reformulation.[26]

We chose 5-methyl-2-[(2-nitrophenyl)amino]-3-thiophenecarbonitrile (**6**), also known as ROY because of the different colors (red, orange, and yellow) exhibited by its various crystalline states. A synthetic precursor of the antipsychotic olanzapine, we envisaged this as an ideal test substrate for polymorph screening using our high-throughput platform. Since the discovery of the first polymorphs of ROY in 1998, it has been the target of numerous experimental and computational studies focusing on new polymorph discovery and prediction. This has resulted in 12 published polymorphs, nine of which have been characterized by X-ray diffraction.[27–30]

In order to screen for new and existing polymorphs of ROY, we undertook a large number of parallel experiments in which nanodroplets of ROY solutions were encapsulated in a selection of inert oils. Individual experiments were checked for crystal growth periodically, and the first single crystals appeared after 12 h.

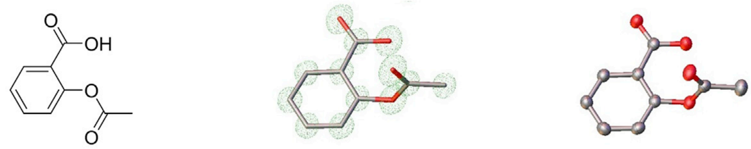

Aspirin (**1**): R_1 = 0.0659, $_{w}R_2$ = 0.1615, Residual e^- density ($e^-Å^3$) min = -0.204 and max = 0.314

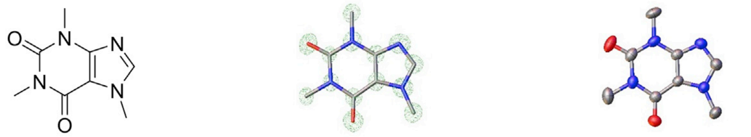

Caffeine (**2**): R_1 = 0.0610, $_{w}R_2$ = 0.1574, Residual e^- density ($e^-Å^3$) min = -0.203 and max = 0.395

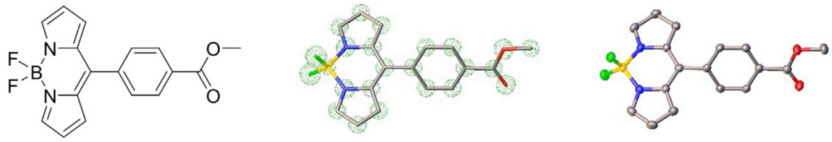

BODIPY (**3**): R_1 = 0.0377, $_{w}R_2$ = 0.0786, Residual e^- density ($e^-Å^3$) min = -0.184 and max = 0.175

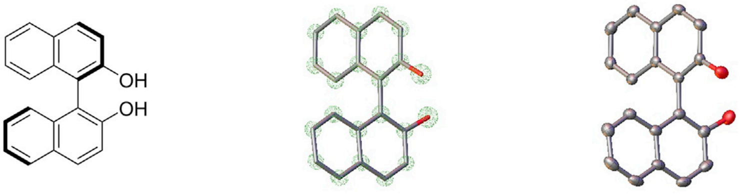

(*R*)-BINOL (**4**): R_1 = 0.0413, $_{w}R_2$ = 0.0839, Residual e^- density ($e^-Å^3$) min = -0.135 and max = 0.143, Flack = 0.08(18)

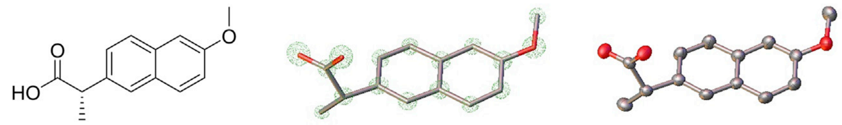

(*S*)-Naproxen (**5**): R_1 = 0.0299, $_{w}R_2$ = 0.0646, Residual e^- density ($e^-Å^3$) min = -0.173 and max = 0.135, Flack = -0.015(7)

Figure 3. Molecular Structures, Electron Density Maps, and Refined Crystallographic Molecular Models Derived from SCXRD Analysis of Single Crystals of Compounds 1–5 Formed via the ENaCt Protocol

Each structure is provided with selected crystallographic information.

Suitable single crystals of ROY were obtained from a range of different experimental conditions.

Four of the known ROY polymorphs (Y, R, ON, and ORP) can be accessed through solution-phase crystal growth; the others arise directly or indirectly only via melt experiments. By using our ENaCt protocol, we were able to grow single crystals suitable for SCXRD analysis for all four of the solution-phase accessible polymorphs, where Y was the most commonly observed (Figure 4).

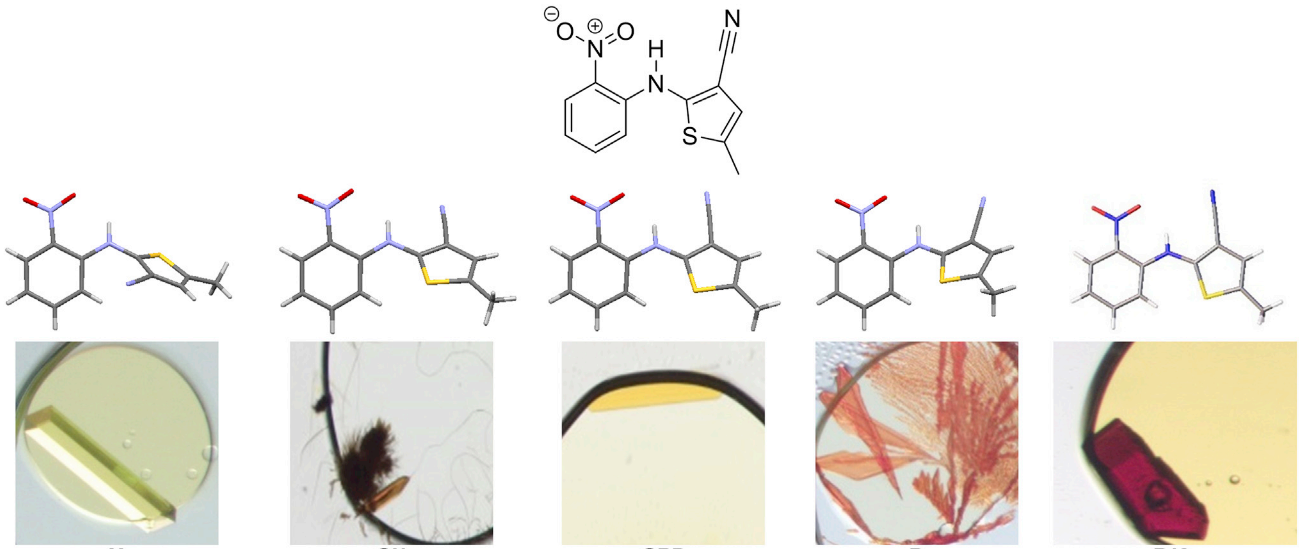

Y	ON	ORP	R	R18
$P2_1/n$, R_1 = 0.0266, θ = 106.1 °	$P2_1/c$, R_1 = 0.0357, θ = -53.8 °	*Pbca*, R_1 = 0.0336, θ = -39.5 °	*P*-1, R_1 = 0.0448, θ = -19.3 °	*P-1*, R_1 = 0.0299, θ = 10.276 ° and 5.009 ° (Z' = 2)

Figure 4. Molecular Structures, Crystal Growth Images, and SCXRD Data for Existing (Y, ON, ORP, and R) and New (R18) Polymorphs of ROY (6)

Additionally, deep-red block-shaped crystals of ROY were observed, and they did not match the color, morphology profile, or unit cell of any of the known ROY polymorphs (Figure 5). Full structural investigation by SCXRD (150 K, in-house X-ray diffractometer) confirmed this as a new ROY polymorph (R18) that had not previously been reported experimentally and as only the second ROY polymorph to have a Z′ value > 1. R18 is the 13th polymorph of ROY to be discovered and the tenth to be fully characterized through SCXRD, returning ROY to the status of world record holder for the most polymorphic organic molecule.

Thus, the discovery of R18 further validates the power of the ENaCt protocol not just as a crystallization method for X-ray analysis but also as a potential polymorph screening tool.

Crystallization of an "Uncrystallizable" Molecule: Dithianon (7)

Finally, we wished to apply our ENaCt methodology to the crystallization of a crystallographically challenging or previously deemed "uncrystallizable" sample. We envisaged that the high-throughput capability of ENaCt would allow for the rapid screening of the diverse crystallization conditions required to ensure the growth of a single crystal suitable for X-ray diffraction analysis of such a material. To this end, we selected the functionalized naphthoquinone dithianon (5,10-dioxo-5,10-dihydronaphtho[2,3-*b*][1,4]dithiine-2,3-dicarbonitrile) (7). Dithianon was first introduced in 1963 as a broad-spectrum foliar fungicide and is still used for controlling fungal infections in commercial agriculture.[31] Dinnebier and co-workers have previously shown the existence of four different polymorphs of dithianon (forms 1–4) via refinement against high-resolution X-ray powder diffraction data sets. However, they did not report a SCXRD data set because of "… the lack of single crystals of sufficient size and quality" as a result of the lack of strong intermolecular interactions in the solid state.[32]

Thus, following our ENaCt methodology, we undertook 384 individual crystallization experiments. We dispensed 200 nL of oils (FC 40, Fomblin YR, PDMSO, or mineral

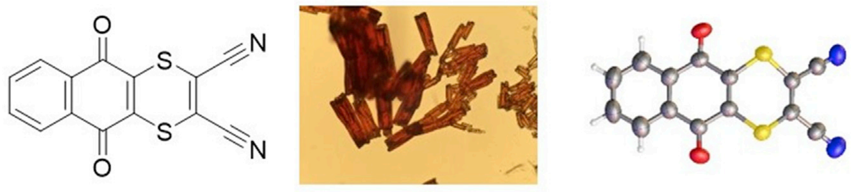

Dithianon (**7**): R_1 = 0.048, $_wR_2$ = 0.1262, Residual e^- density ($e^-Å^3$) min = -0.2 and max = 0.4

200 nL PDMSO, 50 nL of 12.5 mg/mL of dithianon in DMF, 25 nL 2-methyl-2,4-pentanediol.

Figure 5. Molecular Structure, Crystal Growth Image, and SCXRD Data for Dithianon (7), Polymorph 1, Including Selected Crystallographic Information and ENaCt Crystal Growth Conditions

oil), followed by 50 nL of dithianon solution (2–50 mg/mL of DMSO or DMF) and 25 or 50 nL of a secondary solvent (toluene, chlorobenzene, 2-methyl-2,4-pentanediol, or water), into 96-well glass plates. After 14 days at RT, experiments were examined by polarizing optical microscopy. 72 crystalline samples (19% of the total number) exhibiting block-, plate-, and needle-like morphologies were identified, and a suitable block-like crystal was analyzed by SCXRD (Figure 5).

The structural model, derived from the SCRXD data, confirmed that we had successfully obtained a single crystal of dithianon (polymorph 1). The structure suffers from minor disorder components, ascribed to stacking faults within the crystal. Further analysis of the structure within the software package CrystalExplorer[33] indicated the presence of only a small number of weak intermolecular interactions within the structure and no interlayer component of the total energy framework exceeding 15 $kJmol^{-1}$ (Figure S3). We propose that the presence of only weak interactions is the cause of the previously failed dithianon crystallization attempts using traditional methodologies. Thus, these experiments further demonstrate the capability of the ENaCt protocol to address even the most challenging of crystallization problems.

Conclusion

There is a requirement across the molecular sciences for a widely applicable, high-throughput crystallization method that operates on the microgram scale. The ENaCt protocol described here fulfills these requirements as a tailor-made solution to the crystallization problem. Our results—both the successful crystallization of a diverse set of small molecules (including the "uncrystallizable" dithianon) and the exploration of the polymorphs of ROY—provide strong evidence for the potential of ENaCt as a general tool for the crystallization of organic-soluble small molecules. We anticipate that such facile access to high-quality single crystals through the rapid sampling of large areas of the solid-state energy landscape will provide significant future benefits to the molecular science community.

EXPERIMENTAL PROCEDURES

Resource Availability

Lead Contact

Request for further information should be directed to and will be fulfilled by the Lead Contact, Michael J. Hall (michael.hall@newcastle.ac.uk).

Materials Availability

This study did not generate new unique materials.

Data and Code Availability

The accession numbers for the crystallographic data reported in this paper are CCDC: 1944195–1944211 and 1968245. These data can be obtained free of charge from the Cambridge Crystallographic Data Centre at https://www.ccdc.cam.ac.uk/structures.

SUPPLEMENTAL INFORMATION

Supplemental Information can be found online at https://doi.org/10.1016/j.chempr.2020.04.009.

ACKNOWLEDGMENTS

The authors acknowledge support from the Biotechnology and Biological Sciences Research Council and Natural Products Discovery and Bioengineering Network (BB/L013754/1), the N8 Research Partnership, and the Higher Education Innovation Fund for funding; the Engineering and Physical Sciences Research Council for access to X-ray crystallography facilities (EP/F03637X/1); and Diamond Light Source (beamline I19).

AUTHOR CONTRIBUTIONS

M.J.H. and M.R.P. directed and conceived this project; A.R.T., R.R., C.J.M., and P.G.W. conducted the experimental work; S.E.H. contributed statistical analysis; J.W.S. provided ROY and contributed to ROY-related experimental design; and P.T. contributed to robotic experimental design. All authors discussed the results and wrote the manuscript.

DECLARATION OF INTERESTS

A.R.T., M.J.H., and M.R.P. have filed a patent application based on this work: GB1908349.2.

Received: December 2, 2019
Revised: February 5, 2020
Accepted: April 15, 2020
Published: May 7, 2020

REFERENCES

1. Koritsanszky, T.S., and Coppens, P. (2001). Chemical applications of X-ray charge-density analysis. Chem. Rev. *101*, 1583–1627.
2. Flack, H.D., and Bernardinelli, G. (2008). The use of X-ray crystallography to determine absolute configuration. Chirality *20*, 681–690.
3. Howard, J.A.K., and Probert, M.R. (2014). Cutting-edge techniques used for the structural investigation of single crystals. Science *343*, 1098–1102.
4. Johnson, N.T., Waddell, P.G., Clegg, W., and Probert, M.R. (2017). Remote access revolution: chemical crystallographers enter a new era at diamond light source beamline. 19. Crystals *7*, 360.
5. Taylor, R., and Wood, P.A. (2019). A million crystal structures: the whole is greater than the sum of its parts. Chem. Rev. *119*, 9427–9477.
6. Grothe, E., Meekes, H., Vlieg, E., ter Horst, J.H., and de Gelder, R. (2016). Solvates, salts, and cocrystals: a proposal for a feasible classification system. Cryst. Growth Des. *16*, 3237–3243.
7. Spingler, B., Schnidrig, S., Todorova, T., and Wild, F. (2012). Some thoughts about the single crystal growth of small molecules. CrystEngComm *14*, 751–757.
8. Inokuma, Y., Yoshioka, S., Ariyoshi, J., Arai, T., Hitora, Y., Takada, K., Matsunaga, S., Rissanen, K., and Fujita, M. (2013). X-ray analysis on the nanogram to microgram scale using porous complexes. Nature *495*, 461–466.
9. Hoshino, M., Khutia, A., Xing, H., Inokuma, Y., and Fujita, M. (2016). The crystalline sponge method updated. IUCrJ *3*, 139–151.
10. Jones, C.G., Martynowycz, M.W., Hattne, J., Fulton, T.J., Stoltz, B.M., Rodriguez, J.A., Nelson, H.M., and Gonen, T. (2018). The CryoEM method MicroED as a powerful tool for small molecule structure determination. ACS Cent. Sci. *4*, 1587–1592.
11. Gruene, T., Wennmacher, J.T.C., Zaubitzer, C., Holstein, J.J., Heidler, J., Fecteau-Lefebvre, A., De Carlo, S., Müller, E., Goldie, K.N., Regeni, I., et al. (2018). Rapid structure determination of microcrystalline molecular compounds using electron diffraction. Angew. Chem. Int. Ed. *57*, 16313–16317.
12. Jones, C.G., Asay, M., Kim, L.J., Kleinsasser, J.F., Saha, A., Fulton, T.J., Berkley, K.R., Cascio, D., Malyutin, A.G., Conley, M.P., et al. (2019). Characterization of reactive organometallic species *via* MicroED. ACS Cent. Sci. *5*, 1507–1513.
13. Brázda, P., Palatinus, L., and Babor, M. (2019). Electron diffraction determines molecular absolute configuration in a pharmaceutical nanocrystal. Science *364*, 667–669.
14. Nývlt, J. (1995). The Ostwald rule of stages. Cryst. Res. Technol. *30*, 443–449.
15. Alvarez, A.J., and Myerson, A.S. (2010). Continuous plug flow crystallization of

pharmaceutical compounds. Cryst. Growth Des. *10*, 2219–2228.

16. Foster, J.A., Damodaran, K.K., Maurin, A., Day, G.M., Thompson, H.P.G., Cameron, G.J., Bernal, J.C., and Steed, J.W. (2017). Pharmaceutical polymorph control in a drug-mimetic supramolecular gel. Chem. Sci. *8*, 78–84.

17. Chen, C., Cook, O., Nicholson, C.E., and Cooper, S.J. (2011). Leapfrogging ostwald's Rule of Stages: crystallization of stable γ-glycine directly from microemulsions. Cryst. Growth Des. *11*, 2228–2237.

18. Nievergelt, P.P., Babor, M., Čejka, J., and Spingler, B. (2018). A high throughput screening method for the nano-crystallization of salts of organic cations. Chem. Sci. *9*, 3716–3722.

19. Chayen, N.E., Shaw Stewart, P.D., and Blow, D.M. (1992). Microbatch crystallization under oil – a new technique allowing many small-volume crystallization trials. J. Cryst. Growth *122*, 176–180.

20. Chayen, N.E. (1997). The role of oil in macromolecular crystallization. Structure *5*, 1269–1274.

21. Babor, M., Nievergelt, P.P., Čejka, J., Zvoníček, V., and Spingler, B. (2019). Microbatch under-oil salt screening of organic cations: single-crystal growth of active pharmaceutical ingredients. IUCrJ *6*, 145–151.

22. McCullagh, P. (1980). Regression models for ordinal data. J. R. Stat. Soc. B *42*, 109–127.

23. Dolomanov, O.V., Bourhis, L.J., Gildea, R.J., Howard, J.A.K., and Puschmann, H. (2009). OLEX2: a complete structure solution, refinement and analysis program. J. Appl. Crystallogr. *42*, 339–341.

24. Sheldrick, G.M. (2015). Structure determination revisited. Acta Crystallogr. A Found. Adv. *71*, s9.

25. Cruz-Cabeza, A.J., and Bernstein, J. (2014). Conformational polymorphism. Chem. Rev. *114*, 2170–2191.

26. Chemburkar, S.R., Bauer, J., Deming, K., Spiwek, H., Patel, K., Morris, J., Henry, R., Spanton, S., Dziki, W., Porter, W., et al. (2000). Dealing with the impact of ritonavir polymorphs on the late stages of bulk drug process development. Org. Process Res. Dev. *4*, 413–417.

27. Chen, S., Guzei, I.A., and Yu, L. (2005). New polymorphs of ROY and new record for coexisting polymorphs of solved structures. J. Am. Chem. Soc. *127*, 9881–9885.

28. Tan, M., Shtukenberg, A.G., Zhu, S., Xu, W., Dooryhee, E., Nichols, S.M., Ward, M.D., Kahr, B., and Zhu, Q. (2018). ROY revisited, again: the eighth solved structure. Faraday Discuss. *211*, 477–491.

29. Gushurst, K.S., Nyman, J., and Boerrigter, S.X.M. (2019). The PO13 crystal structure of ROY. CrystEngComm *21*, 1363–1368.

30. Nyman, J., Yu, L., and Reutzel-Edens, S.M. (2019). Accuracy and reproducibility in crystal structure prediction: the curious case of ROY. CrystEngComm *21*, 2080–2088.

31. Vidhyasekaran, P. (2004). Concise Encyclopedia of Plant Pathology (Food Products Press).

32. Halasz, I., Dinnebier, R., Chiodo, T., and Saxell, H. (2012). Structures of four polymorphs of the pesticide dithianon solved from X-ray powder diffraction data. Acta Crystallogr. B *68*, 661–666.

33. Mackenzie, C.F., Spackman, P.R., Jayatilaka, D., and Spackman, M.A. (2017). CrystalExplorer model energies and energy frameworks: extension to metal coordination compounds, organic salts, solvates and open-shell systems. IUCrJ *4*, 575–587.

Chem, Volume *6*

Supplemental Information

Encapsulated Nanodroplet Crystallization of Organic-Soluble Small Molecules

Andrew R. Tyler, Ronnie Ragbirsingh, Charles J. McMonagle, Paul G. Waddell, Sarah E. Heaps, Jonathan W. Steed, Paul Thaw, Michael J. Hall, and Michael R. Probert

Figure S1: Additional compounds **S1-S4** for which single crystals were successfully grown *via* the ENaCt protocol and subsequently analysed by SCXRD. Molecular structures, electron density maps and refined crystallographic molecular models are shown. Each structure is provided with selected crystallographic information and ENaCt conditions for crystal growth.

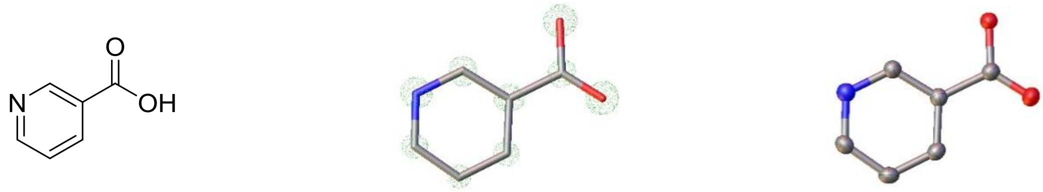

Nicotinic acid (**S1**): R_1 = 0.0594, $_wR_2$ = 0.1100, Residual e^- density ($e^-Å^3$) min = -0.231 and max = 0.1171
200 nL Mineral oil, 50 nL of 40 mg/mL of nicotinic acid in DMSO, 100 nL toluene.

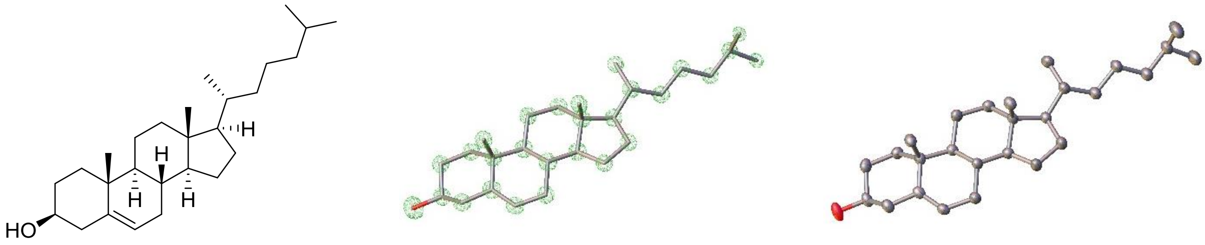

Cholesterol (**S2**): R_1 = 0.0594, $_wR_2$ = 0.1118, Residual e^- density ($e^-Å^3$) min = -0.162 and max = 0.199
200 nL FC-40, 50 nL of 25 mg/mL of cholesterol in DMF, 25 nL *n*-butanol.

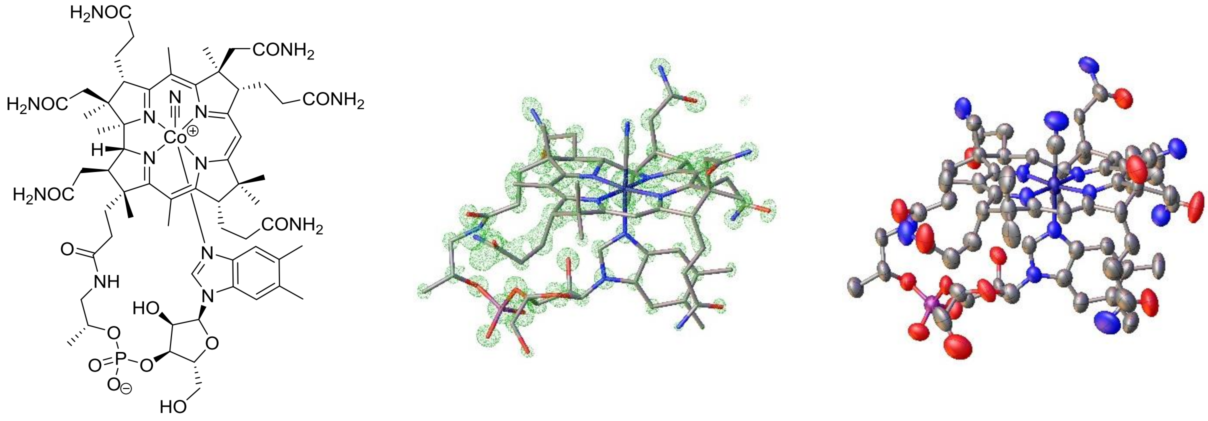

Vitamin B12 (**S3**): R_1 = 0.0902, $_wR_2$ = 0.1535, Residual e^- density ($e^-Å^3$) min = -0.433 and max = 0.260, Flack = 0.071(12)
200 nL Mineral oil, 50 nL of 50 mg/mL in DMSO, 50 nL (±)-2-methyl-2,4-pentanediol.

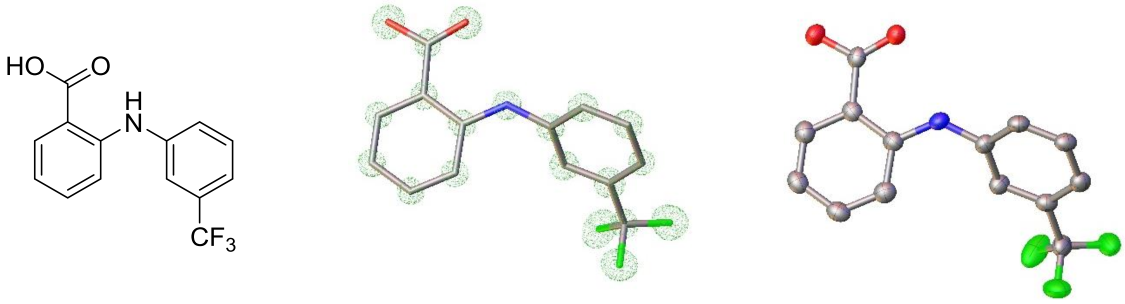

Flufenamic acid (**S4**): R_1 = 0.1093 , $_wR_2$ = 0.1776, Residual e^- density ($e^-Å^3$) min = -0.314 and max = 0.343
200 nL PDMSO, 50 nL of 50 mg/mL flufenamic acid in DMF, 50 nL H_2O.

Figure S2: Additional compounds **S5-S7** for which single crystals were successfully grown *via* the ENaCt protocol and subsequently analysed by SCXRD. Molecular structures, electron density maps and refined crystallographic molecular models are shown. Each structure is provided with selected crystallographic information and ENaCt conditions for crystal growth.

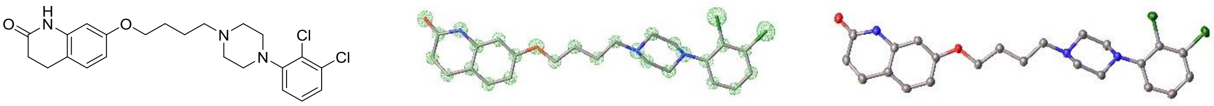

Aripiprazole (**S5**): R_1 = 0.0599, $_wR_2$ = 0.0947, Residual e^- density ($e^-Å^3$) min = -0.256 and max = 0.250
200 nL Fomblin Y, 50 nL of 20 mg/mL of aripiprazole in DMSO.

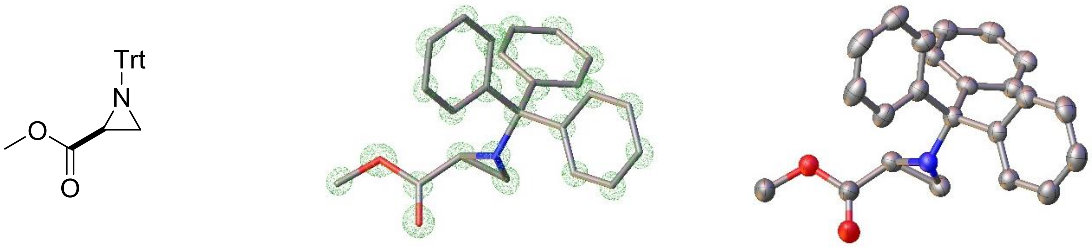

Methyl (*S*)-1-tritylaziridine-2-carboxylate (**S6**): R_1 = 0.0461, $_wR_2$ = 0.0935, Residual e^- density ($e^-Å^3$) min = -0.129 and max = 0.130, Flack = 0.0(2)
200 nL PDMSO, 50 nL of 40 mg/mL of methyl (*S*)-1-tritylaziridine-2-carboxylate in DMF, 100 nL H_2O.

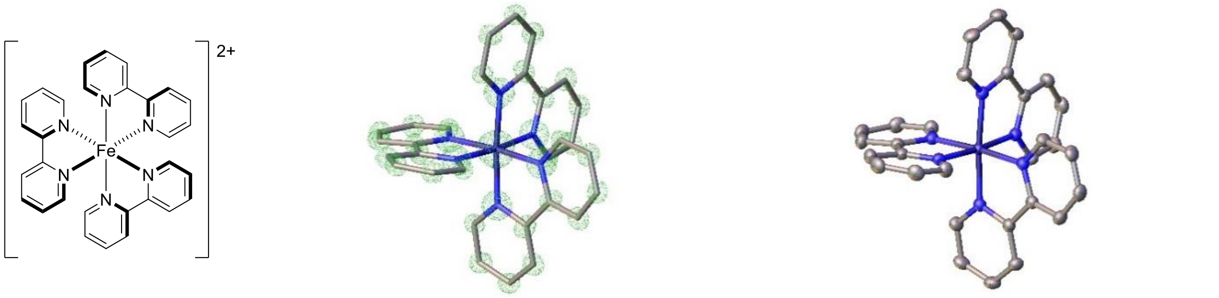

$[Fe(bipy)_3]^{2+}\cdot 2PF_6^-$ (**S7**): R_1 = 0.0331, $_wR_2$ = 0.0707, Residual e^- density ($e^-Å^3$) min = -0.288 and max = 0.299
200 nL FC 40, 50 nL of 25 mg/mL of $[Fe(bipy)_3]^{2+}\cdot 2PF_6^-$ in DMSO (*Note:* counter ions omitted for clarity).

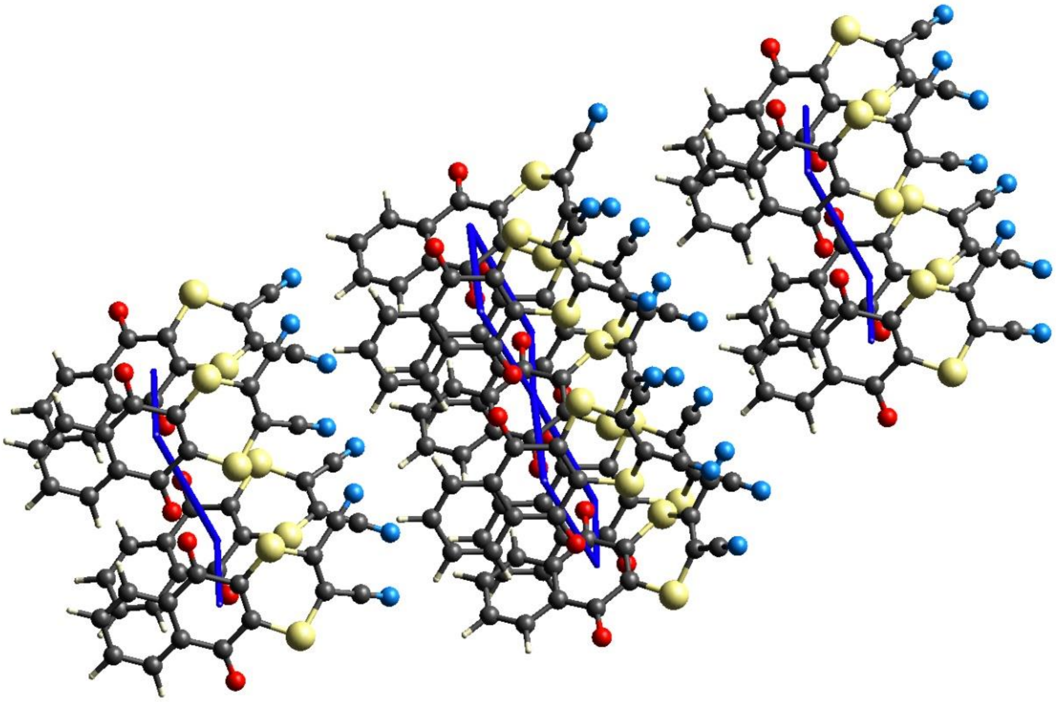

Figure S3: Total-energy-framework diagram for dithianon using cylinder size 50 for energies. Note energies less than 15 kJmol^{-1} have been omitted for clarity. Energies were calculated for the 150 K structure obtained by single crystal X-ray diffraction using the B3LYP/6-31G(d,p) functionals.

Supplemental Experimental Procedures

Materials.

Commercially available oils and chemical substrates were purchased from Sigma Aldrich, Fluorochem, Alfa Aesar or Molecular Dimensions.
5-Methyl-2-[(2-nitrophenyl)amino]-3-thiophenecarbonitrile (ROY) was provided by J.W. Steed, metal complex ($[Fe(bipy)_3]^{3+}$) was provided by M.R. Probert, methyl (*S*)-1-tritylaziridine-2-carboxylate was provided by A.R. Tyler, and methyl 4-(5,5-difluoro-5*H*-4λ^4,5λ^4-dipyrrolo[1,2-*c*:2',1'-*f*][1,3,2]diazaborinin-10-yl)benzoate was provided by M.J. Hall.
Laminex™ glass 96 well plates with a 100 micron spacer and cover slips were purchased from Molecular Dimensions. Glass coated 96 well plates were obtained from Fisher Scientific.

Sample preparation

Stock solutions of each substrate were freshly prepared for each crystallisation experiment. Samples were weighed into a screw top vial, dissolved in a suitable solvent at the desired concentration and then warmed where appropriate (40 °C, 30 min) to ensure that all solids were dissolved. Stock solutions were left to cool (1 hour, r.t.) and assessed *via* optical microscopy to ensure premature crystallisation had not occurred before being used in any ENaCt experiment.

Crystallisation of substrates

Crystallisation experiments were completed using a TTP LabTech mosquito® liquid handling robot using LAMINEX™ glass 96 well plates with a 100 micron spacer and sealed with a glass cover slip. An appropriate volume (typically 200 mL) of each oil was first dispensed onto a LAMINEX™ plate (aspirate 1.0 mm/min, dispense 1.0 mm/min). After which 50 nL of substrate solution was injected into each oil droplet (aspirate 20 mm/min, dispense 20 mm/min), followed by an additional injection of a secondary solvent if required (aspirate 20 mm/min, dispense 20 mm/min). Plates were then sealed with a glass cover slip, stored in the dark at room temperature and inspected for crystal growth at regular intervals. Visualisation of the experiment wells was carried out with a Nikon SMZ1000 microscope fitted with a cross polariser. Photographs were taken with a GXCAM-U3-5 5.1MP camera.

Single crystal X-ray diffraction studies

Upon observation of suitable crystals, wells were opened, with use of a tungsten carbide scriber to remove a small portion of the glass cover slide, and the crystal manipulated using Mitegen Kapton microtools. Crystals were transferred to a glass slide and extracted under oil (Fomblin YR-1800) with a standard Mitegen Kapton loop, and mounted onto either an in-house diffractometer (Rigaku Oxford Diffraction Xcalibur, Atlas, Gemini ultra, or Bruker D8 Vantage, Photon 2, dual Incoatec IµS (Ag/Cu) both equipped with an Oxford Cryosystems Cryostream open-flow cooling device and maintained at 150 K) or stored in a liquid N_2 storage dry shipper for investigation on beamline I19 of Diamond Light Source, *via* remote access.

Ordinal logistic regression

Let the outcome of each experiment be encoded by an ordinal variable Y where:

Y = {1, if no solid (still solvated); 2, if phase separation; 3, if solid, but not crystalline; 4, if small crystal; 5, if large crystal.

A proportional odds model for ordinal logistic regression was used to assess the relationship between the response variable Y and covariates, which comprised two quantitative variables (volume of solvent and volume of oil) and three categorical variables (solvent, type of oil and compound). Inference was performed in the Bayesian framework, carrying out the necessary numerical computation using rstan[S1], the R interface to the Stan software[S2]. Use of FC40 and, in particular, PDMSO both appear to have a positive relationship with the response variable; the posterior probabilities that the corresponding regression coefficients are greater than zero, indicating positive association, are each equal to 1.00, to two decimal places.

Supplemental References

S1. Stan Development Team (2016). RStan: the R interface to Stan. R package version 2.12.1.

S2. Carpenter, B., A. Gelman, M. D. Hoffman, D. Lee, B. Goodrich, M. Betancourt, M. A. Brubaker, J. Guo, P. Li and A. Riddell (2017). Stan: A probabilistic programming language. *Journal of Statistical Software* 76 (1), 1–32.
